# The *Staphylococcus aureus* cardiac snake

**DOI:** 10.1007/s12471-023-01790-3

**Published:** 2023-07-06

**Authors:** Gijs J. van Steenbergen, Willemijn Tunnissen, Naomi Timmermans, Patrick Houthuizen, Rene van den Broek, Thomas van Brakel

**Affiliations:** 1 Department of Cardiothoracic Surgery, Catharina Hospital, Eindhoven, The Netherlands; 2 Department of Cardiology, Catharina Hospital, Eindhoven, The Netherlands; 3 Department of Anaesthesiology, Catharina Hospital, Eindhoven, The Netherlands

**Fig. 1:**
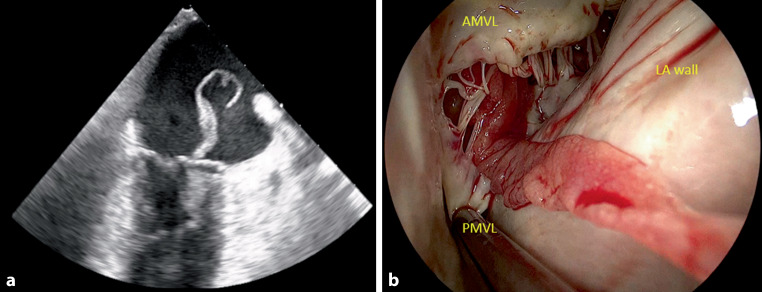
Image of the vegetation on transoesophageal echocardiography moving freely between the left atrium and left ventricle (**a**, Video 1). Intraoperative inspection of the mitral valve revealed that the vegetation originated from the subvalvular apparatus of the mitral valve and had damaged segment P1 of the posterior leaflet (**b**). The vegetation was removed en bloc (Video 2) and due to the damaged posterior leaflet, the mitral valve was replaced with a biological prosthesis. *AMVL* anterior mitral valve leaflet, *PMVL* posterior mitral valve leaflet, *LA* left atrium

A 73-year-old male presented to the emergency department with delirium and fever (39.2 °C) 1 week after receiving an intra-articular corticosteroid injection in his left glenohumeral joint to treat bursitis. Janeway lesions were noticed. The cardiologist was consulted and performed transthoracic and transoesophageal echocardiography, on which an impressive 8‑cm snake-like vegetation was seen on the mitral valve with minor regurgitation (Fig. 1a; see also Video 1 in Electronic Supplementary Material). Blood cultures grew *Staphylococcus aureus*. Based on the modified Duke criteria (two major criteria and three minor criteria), definite endocarditis was diagnosed [1]. Given the size and mobility of the vegetation, emergent cardiac surgery was performed, in which the vegetation was removed en bloc and the mitral valve was replaced (Fig. 1b; see also Video 2 in Electronic Supplementary Material). The patient died on the 17th postoperative day as a result of uncontrolled infection. Swift diagnosis and treatment in patients with (suspected) *S. aureus* endocarditis is crucial to improve outcomes.

### Supplementary Information

Transoesophageal echocardiogram of the vegetation

Findings during surgery
